# Adaptive Petal Reflector: In-Lab Software Configurable Optical Testing System Metrology and Modal Wavefront Reconstruction

**DOI:** 10.3390/s23177316

**Published:** 2023-08-22

**Authors:** Carl Johan G. Nielsen, André Preumont

**Affiliations:** Department of Control Engineering and System Analysis, Université Libre de Bruxelles (ULB), 1050 Brussels, Belgium

**Keywords:** spherical petal reflector, deployable reflector, surface figure metrology, SCOTS, Shack–Hartmann, modal reconstruction, vibration modes

## Abstract

This paper addresses two aspects of the metrology of spherical, petal polymer reflectors which are part of an effort by the European Space Agency (ESA) to develop actively controlled foldable reflectors, enabling larger apertures on CubeSats and small satellites. The first problem is that of measuring the surface figure error of the spherical reflector alone during the development phase, and to assess the quality before assembling the telescope (large stroke, low accuracy). The SCOTS (Software Configurable Optical Testing System) appears to provide a fast and satisfactory solution to this problem. The second problem is the wavefront error reconstruction when the petal reflector is mounted on the telescope, because parts of the petals are obscured by the secondary mirror, in such a way that the petals appear completely disconnected, making the gradient-based metrology impossible. Using the fact that the petals have common mechanical boundary conditions at the central support ring, the problem is solved by using a set of orthogonal modes satisfying the same boundary conditions. The vibration modes are used for this purpose; the modal amplitudes are reconstructed from slope data outside the obstruction, allowing for wavefront error reconstruction over the entire surface.

## 1. Introduction

**Figure 1:**
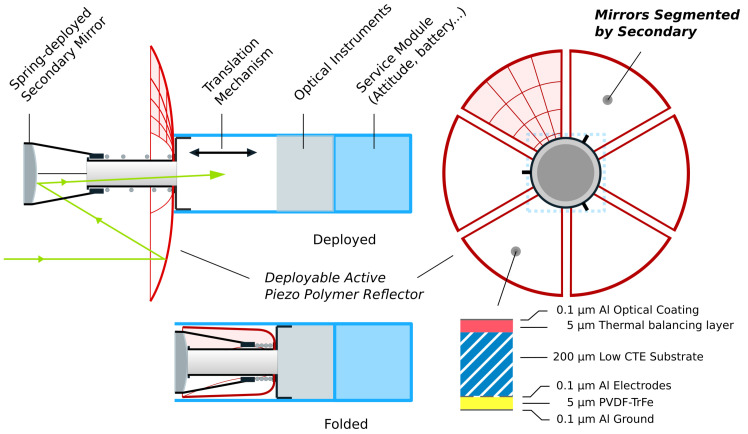
Illustration of the system design of the active petal reflector in folded and deployed states. The reflector (red) is coated with a piezoelectric material, allowing for active control in closed loop given a metrology system. Since the secondary will obstruct the primary mirror, the recovered sensor data will be segmented, and special wavefront reconstruction is needed to recover the error accurately.

This study is motivated by the ongoing, ESA-funded development of foldable, polymer, actively controlled petal reflectors for CubeSat applications, as seen in Figure 1 [1,2,3]. As the reflector is foldable, larger apertures are enabled on small satellite platforms. Upon unfolding, active control is attained by a thin layer of piezoelectric PVDF-TrFE between aluminum electrodes, allowing for an accurate surface to be maintained. Two problems are addressed in the development and operation of the active reflector, namely:

- The developer of the reflector must have access to a metrology system capable of a rapid evaluation of the manufacturing process, and to verify, without the availability of the whole telescope, that the surface figure aberration will remain within the envelope that can be corrected subsequently by the control system.
- When the primary reflector is mounted on the telescope, part of the petals are in the shade of the secondary mirror and they appear as segments (i.e., independent of each other). However, unlike segmented mirrors which require edge sensors, all the petals have a common mechanical boundary condition at the inner ring of the reflector. This paper investigates the use of this property to implement a wavefront reconstruction based on the measurement of the x-y slopes at a set of grid points (Shack–Hartmann-type sensor).

Other concepts for deployable space telescopes for CubeSats include rigid segmented (hinged) reflectors [4] and photon sieve designs [5]. In contrast, the folding of the reflector described herein is attained by its own flexibility. Further ESA R&D activities in optical design and control of space telescopes are reviewed in [6,7].

### 1.1. In-Lab Metrology

For the metrology in a lab environment, a technique is needed which is able to provide a few-micron accuracy, sub-micron repeatable test, which at the same time has sufficient dynamic range to capture aberrations larger than 100μm Peak-to-Valley (PV). While interferometric techniques would provide accuracy much better than required, the dynamic range would be challenging. Conversely, techniques such as photogrammetry would have sufficient range, but the accuracy would be challenging. The Software Configurable Optical Testing System (SCOTS) was developed specifically to overcome similar challenges in testing solar concentrators [8]. The SCOTS test has since been progressively refined, and some implementations are successfully measuring sub-nm errors on X-ray mirrors [9]. The test is based on deflectometry and requires three components: a reflecting surface under test, a screen that projects a series of fringe patterns with sinusoidal intensity, and a camera recording the distorted fringes as reflected by the mirror. A main difficulty in obtaining high-precision results is the placement of the camera, screen, and mirror components, and high accuracy requires measurements of the relative positions with, e.g., laser trackers [10]. For the purposes of our work, such strict geometric control will not be necessary due to the relatively relaxed accuracy requirements; the purpose is to test that the manufacturing error is within the actuator stroke.

One challenge with the SCOTS test is that results are obtained easily, but corroborating them with reality is more difficult. One approach is to use a “closed-loop” system in which the resulting geometry from the measurement is introduced into a ray-tracing software, allowing the user to see if the experimentally recorded raw image data correspond to simulated image data. This approach will be used in this paper, and a similar approach is reported in [11]. Unique to this research is the measurement and reconstruction of petal geometries from measured slope data, where specialized algorithms for phase unwrapping and shape integration are needed to handle discontinuities in the reflector. The methods developed in this article would also be extensible to other topologies and have been made available as open source [12].

### 1.2. Vibration Modal Wavefront Reconstruction

Once mounted on the telescope, the adaptive petal reflector is intended to be controlled by an array of piezoelectric actuators. This assumes the availability of a wavefront sensor. Several approaches exist to wavefront sensing and reconstruction, for instance zonal [13] or modal (Zernike, Legendre or Chebyshev [14,15]) reconstruction techniques for Shack–Hartmann sensors, neural network-based reconstruction for pyramid wavefront sensors [16] or even wavefront sensorless adaptive optics [17]. In this work, we focus on gradient data obtained from, e.g., a Shack–Hartmann sensor, and the reconstruction of the wavefront from this data.

Due to the petal geometry, the central part of the reflector is obscured by the shade of the secondary mirror (Figure 1), in such a way that the petals appear disconnected (as segments), making the gradient-based metrology impossible. However, since the petals have common mechanical boundary conditions at the inner ring of the reflector, this feature can be exploited to implement a modal reconstruction method based on a complete set of orthogonal modes satisfying the same boundary conditions. The normal vibration modes [18,19] appear as a natural choice. Having reconstructed the modal amplitudes from the slope data in the accessible part of the reflector, an approximation of the wavefront aberration over the entire reflector is reconstructed from the modal expansion. It is worth noting that the only strict requirement on the vibration modes is that they satisfy the geometric boundary conditions at the inner ring of the reflector. Two sets of modes will be compared below: the modes of the spherical petal shell constituting the reflector and the modes of a uniform plate with the same profile.

## 2. SCOTS

In a SCOTS setup, successive images of sinusoidal fringes (phase screens) are displayed on a monitor. The images of the phase screens are then reflected by the mirror and captured by the camera. This allows the shape of the mirror to be reconstructed from the acquired camera images, as the slope at a given location on the mirror can be recovered from the distorted images. The points on the mirror in which the slope is reconstructed are referred to as the Mirror Pixels.

**Figure 2:**
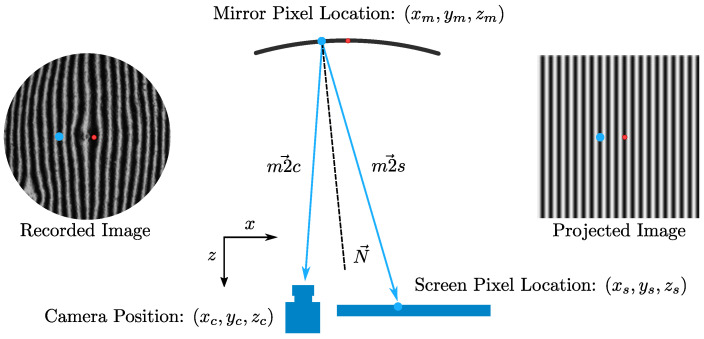
Illustration of the SCOTS test. The two vectors $\vec{m2c}$ and $\vec{m2s}$ together describe the surface slope. The blue dot illustrates how the recorded image relates a position of the Mirror Pixel to its corresponding screen location. The red dot shows the zero-phase location. Several images are needed to accurately relate the projected and recorded images in terms of phase.

To understand the test, consider a Mirror Pixel with location coordinates $\left(x_{m},y_{m},z_{m}\right)$ and a pinhole camera with location coordinates $\left(x_{c},y_{c},z_{c}\right)$. Tracing (in reverse) the ray of the camera through its reflection with the mirror leads to a single screen point $\left(x_{s},y_{s},z_{s}\right)$. The goal of SCOTS is the accurate identification of the two geometric vectors for each mirror pixel: the Mirror to Camera vector ($\vec{m2c}$) and the Mirror to Screen vector ($\vec{m2s}$). An illustration of the SCOTS test can be seen in Figure 2.

Given these vectors, the surface vector normal to the mirror, $\vec{N}$, is obtained as the sum of the normalized vectors:

(1) $$\vec{N}=\frac{\vec{m2c}}{\left|\right|\vec{m2c}\left|\right|}+\frac{\vec{m2s}}{\left|\right|\vec{m2s}\left|\right|}$$

From the surface normal vector $\vec{N}$, the slope of the mirror $\left(w_{x},w_{y}\right)$ can then be calculated:

(2) $$\vec{n}=\frac{\vec{N}}{\vec{\left||N|\right|}}$$

(3) $$w_{x}=\frac{-{\vec{n}}_{x}}{{\vec{n}}_{z}}$$

(4) $$w_{y}=\frac{-{\vec{n}}_{y}}{{\vec{n}}_{z}}$$

The surface of the mirror can subsequently be reconstructed from the slope data. The challenge lies in the accurate identification of these geometric parameters for each mirror pixel. The position of the camera aperture can be measured directly. Furthermore, the position of any pixel on the screen can be determined by choosing an origin and measuring the screen pixel scale. Likewise, under the assumption that the camera creates a flat image, the location of Mirror Pixels may be found by selecting an origin and measuring its location along with the image pixel scale.

In the simplest possible implementation, a single Screen Pixel may be lit at a time. In this case, all nine geometric parameters of Figure 2 are known by simple measurement, and the slope can be calculated as shown. However, for a screen with millions of pixels, this is tedious. Alternatively, the entire screen can be used to project a vertical and horizontal sinusoidal fringe map, such that each point on the screen has two associated unique phase values. These unique phase values must then be recovered from the recorded image, allowing every Mirror Pixel to have an associated screen location. The process of recovering the phase happens in three steps:

- **Zero-phase recovery**: A single pixel is lit up in the (0,0)-phase location of the screen and its position in the recorded image is saved (Figure 2, red dot).
- **Determination of phase**: Successive horizontal and vertical sinusoidal fringes with varying phase offsets are displayed. For each individual Mirror Pixel, a sine function is fit on the data from the images to determine the phase value of this pixel from −π to π (Figure 2, blue dot).
- **Phase unwrapping**: as the previous step happened on a per-pixel basis, there is no global information about the phase. The global phase is recovered by a numerical phase unwrapping technique and offset according to the previously recovered zero-phase location.

With the phase values known in both the recorded image and the projected image, all nine geometric parameters can be inferred, and the slope may be calculated. The process of finding the geometric parameters is formalized in Appendix A. Finally, the slopes may be integrated to find the shape. This step usually happens by the integration of the difference between the recorded and expected slope, rather than directly from the slope.

### 2.1. Raytracing

**Figure 3:**
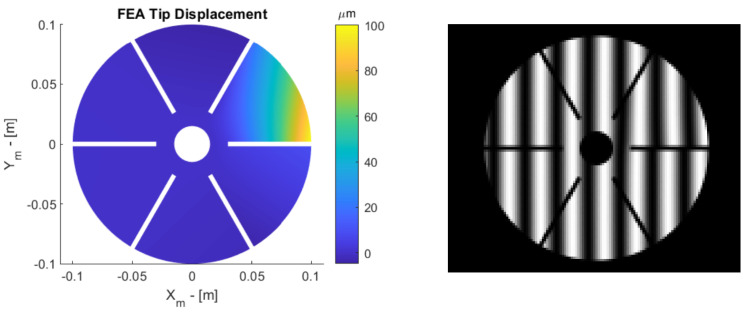
(**left**) A 100−micron tip displacement aberration simulated with FEA. (**right**) The resulting ray-traced image from the simulation. Note that, on the right side, the fringes do not line up exactly due to the tip displacement.

Before using the SCOTS test to measure a new topology, its important to verify the test. One approach to this is to simulate images using raytracing in a known virtual environment. To do this, a 3D model of the petal with a known aberration may be loaded into a raytracing software. The camera can be modeled as a pinhole, and the screen is an emissive source of varying intensity. In the raytracing software, the camera launches rays “in reverse” toward the 3D model; they are then reflected and intersect the emissive source (screen). The value of the emissive source is sampled to generate the image. The software used is Raysect [20] in Python, which can be found from the Matlab environment. An example aberration of the petal reflector is illustrated in Figure 3 (**left**) in which a 100 µm tip displacement is applied. The resulting ray-traced image can be seen in Figure 3 (**right**). The tip displacement is generated by applying a fixed displacement in an FEM software, ensuring the displacement is representative of expected deformations on the reflector. Notable parameters are as follows: radius of curvature—2.5 m; diameter—20 cm; camera location $\left(x_{c},y_{c},z_{c}\right)$—(−50 mm,0,0); screen origin $\left(x_{s},y_{s},z_{s}\right)$—(50 mm,0,0); mirror origin $\left(x_{m},y_{m},z_{m}\right)$—(0, 0,−1400 mm).

**Figure 4:**
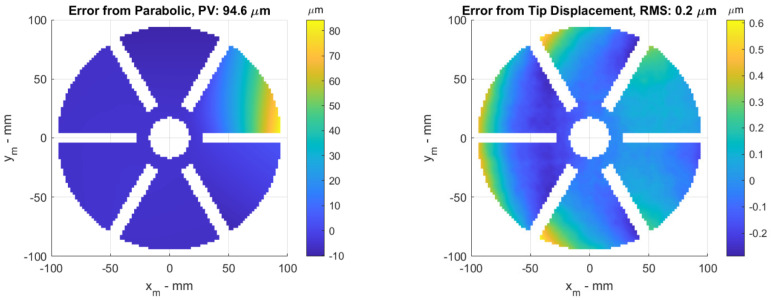
(**left**) Reconstructed aberration from simulated images of 100−micron tip displacement. The geometry is slightly eroded to avoid edge problems. This also reduces the apparent PV slightly. (**right**) Error between the nominal aberration of Figure 3 (**left**) and the measured aberration of Figure 4 (**left**). The main discrepancy arises from the assumption of zero curvature on the primary mirror.

A set of images with horizontal and vertical fringes is generated, and the phase information is captured and reconstructed using a weighted phase unwrapping technique [21,22] where the petal geometry is used as a mask. The shape is then reconstructed from the slope information using the discontinuity-preserving Mumford Shah integration technique [23,24]. The reconstructed aberration can be seen in Figure 4 (**left**), and the error between reconstruction and nominal aberration can be seen in Figure 4 (**right**). The main component of the error shown is from the assumption that the reflector is flat from the point of view of the camera. This assumption leads to a coma error in the case of a full reflector, but the petal integration leads to a more complex error. With this error known, it may be possible to subtract it in the slope calculation for a specific setup, but the accuracy shown is sufficient for our purposes: characterizing different manufacturing techniques and ensuring the resulting errors are within the control stroke of the adaptive reflector.

With the ray-tracing setup, geometric tolerances can also be simulated for expected deviations. Ordinary machine shop measurement tools are used to place the screen and camera with expected tolerances of 2 mm and 2 degrees. Simulations are then made in which the camera is moved in the ray-tracing software, but the input to the reconstruction remains the same. The results are shown in Table 1. Similar deviations are expected from moving the screen or mirror. The errors reported here are higher than in the previous literature [8], with various possible explanations such as the geometric positions of the test components or the influence of the tested aberration.

### 2.2. Experimental Results

**Figure 5:**
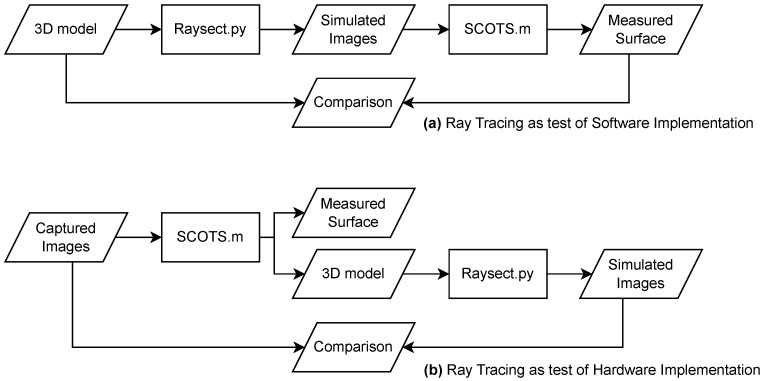
Illustration of the different operating modes of the software. (**a**) The raytracing can be used to test the software implementation by comparing to a known aberration. (**b**) the ray-tracing can be used to verify the hardware implementation by reconstructing a virtual image from the measured surface.

By using the ray-tracing software and comparing the expected deformation with the reconstructed deformation, we confirmed our software implementation of SCOTS works as expected even for the petal geometry. The next step would be to use the implemented software to measure samples. In this case, it is also helpful to have a verification of the hardware setup. As illustrated in Figure 5, this can also be performed using ray-tracing. A series of images may be captured experimentally, and passed to the SCOTS implementation to reconstruct a 3D model of the measured geometry. The 3D model may then be passed to the ray-tracer, simulating an image. The simulated image may then be overlaid with the real image. If the images match accurately, then no major errors are present in the reconstruction.

**Figure 6:**
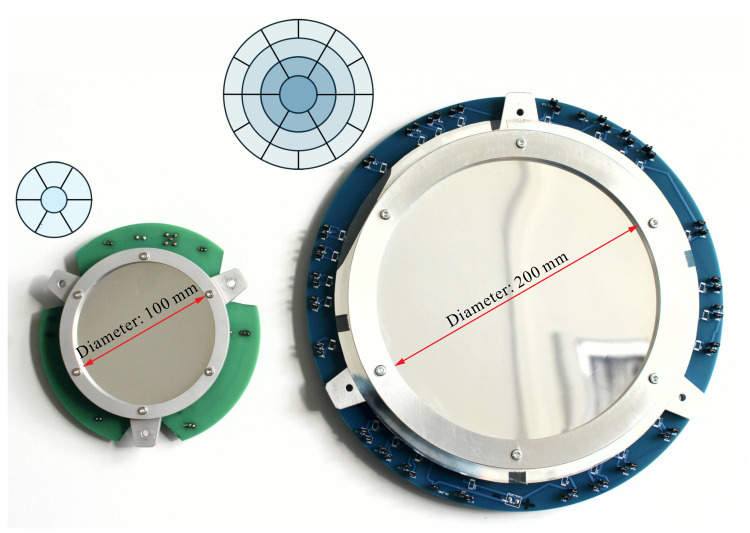
Demonstrators of the previous project [3] with individual electrode control.

**Figure 7:**
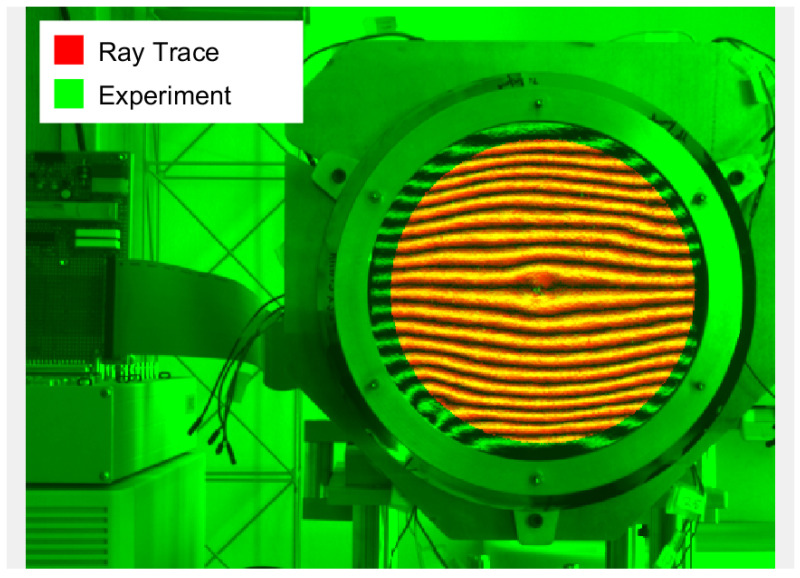
Image overlay of the real image (green) and ray-traced simulation of the measured geometry (red). The two images closely match, showing that the geometry reported by the SCOTS implementation corresponds closely to the real world.

Figure 6 shows two demonstrators manufactured by MateriaNova in the context of the previous project (MATS). The demonstrators consist of a curved polymer shell with spray-coated PVDF-TrFE between vacuum-deposited patterned electrodes, allowing for active control (see also Figure 1). The SCOTS implementation is tested on a similar sample (aluminum-coated polymer only) with 20 cm diameter and 2.5 m radius of curvature. The simulated image and real image are compared in Figure 7. This confirms a close match between the experimental setup and the virtual experiment. Thus, we expect that the tolerances reported in Table 1 are representative of the actual accuracy of the test. With this, we have laid the necessary ground work for measuring deployable petal reflectors in the laboratory.

## 3. Modal Wavefront Reconstruction

**Figure 8:**
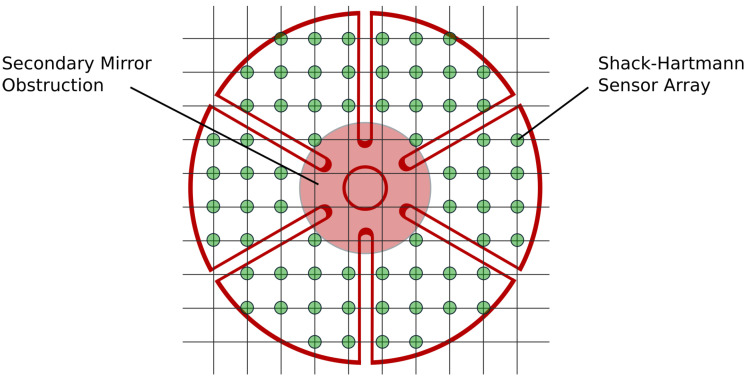
Petal reflector, Shack–Hartmann sensor array, and secondary mirror obscuration. The petals appear disconnected, like on a segmented mirror.

Consider the petal reflector of Figure 8 provided with a micro-lens sensor array (Shack–Hartmann) providing the average x-y slopes at the control points. Due to the obscuration of the secondary mirror, the petals appear disconnected, suggesting that the primary reflector should be considered as segmented. However, the petals have an interesting property that segmented mirrors do not have: they share the same mechanical boundary conditions at the inner support ring of the reflector. This property will be used to reconstruct an approximation of the aberration over the whole reflector surface from slope data.

### 3.1. Vibration Modes

**Figure 9:**
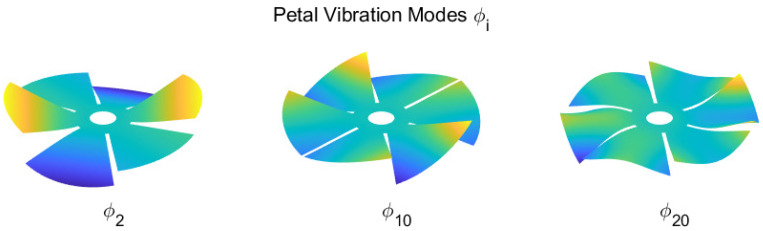
Example of vibration modes of the petal reflector, which will be used in the reconstruction of the aberration.

Let us consider the vibration modes $\phi_{i}$ (Figure 9) of the petal reflector. They constitute a complete set of orthogonal functions, solutions of the eigenvalue problem

(5) $$\left(K-\omega_{i}^{2}M\right)\phi_{i}=0$$

where *K* and *M* are, respectively, the stiffness and the mass matrices of a finite element (FE) model of the structure (the spherical shell petal reflector in this case) and $\omega_{i}$ are the natural frequencies. The mode shape amplitudes $\phi_{i}$ contain the numerical values of the degrees of freedom (d.o.f.) of the finite element model. Using a Guyan reduction [18,19], the degrees of freedom can be selected to contain only the z-components of the nodal displacements of the FE model. Equation (5) does not define the amplitude, but only the shape of the modes; it is customary to normalize them in such a way that $\phi_{i}^{T}M\phi_{i}=1$ (unit modal mass). Figure 9 shows a few examples of the vibration modes of the petal reflector; they all satisfy the mechanical boundary conditions; their shapes have increasing complexity as the order increases. They satisfy the orthogonality condition $\phi_{i}^{T}M\phi_{j}=0$ if (i≠j).

Using the matrix notation $\Phi =(\phi_{1},\phi_{2},\ldots ,\phi_{n})$, the orthogonality condition reads

(6) $$\Phi^{T}M\Phi =I$$

Any surface figure error w may be expanded in the mode shapes

(7) w=Φa

The modal components a are easily obtained from the condition of orthogonality:

(8) $$a=\Phi^{T}Mw$$

**Figure 10:**
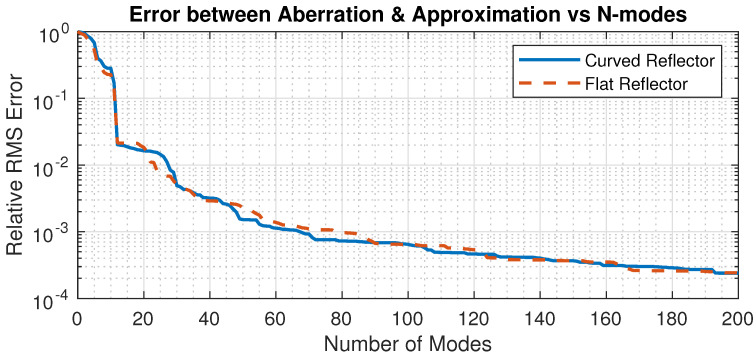
Evolution of the relative RMS residual error as a function of the number of modes in the expansion. Comparison between the mode shapes of the petal spherical shell and the corresponding petal plate. The surface figure error is a tip displacement of 100 µm in the corner of one petal.

The accuracy of the modal expansion increases with the number of modes, as illustrated in Figure 10; the assumed surface figure error is a tip displacement of 100 µm in the corner of one petal; the reflector has a diameter of 30 cm and a radius of curvature of 0.85 m. Triangular Mindlin elements in FEM solver SAMCEF are used, with 3066 nodes in the Guyan reduction. The figure shows the relative RMS residual error as a function of the number of modes *n* included in the expansion. One sees that the residual error reduces rapidly as *n* increases.

Note that the set of modes used in the modal expansion, as seen in Equation (7), does not have to be the modes of the reflector itself, but they must have the same mechanical boundary conditions at the inner support ring. To illustrate this, Figure 10 also shows the relative RMS residual error when the mode shapes of a petal flat plate (with the same inner ring support and outer contour) are used instead of the spherical shell.

### 3.2. Jacobian

The Shack–Hartmann (S-H) sensor is one of the most popular wavefront sensors; it measures the average slopes $\left(s_{x},s_{y}\right)$ of the wavefront at an array of discrete points corresponding to the various sub-apertures of the lenslet array. The total number of sensor outputs is twice the number of active lenslets, $n_{s}$. Such a sensor alone is not appropriate for segmented mirrors, because it cannot detect relative rigid body displacements between segments along *z*. However, for the petal reflector with central obscuration considered here, the restriction is alleviated due to the common support condition at the inner ring.

Taking the partial derivative of Equation (7) with respect to *x* and *y* at the Shack–Hartman sensor points,

(9) $$\begin{array}{c} \left\{\begin{array}{c} w_{x}=\;\Phi_{x}\;a\\ w_{y}=\;\Phi_{y}\;a \end{array}\right. \end{array}$$

where $\Phi_{x}$ and $\Phi_{y}$ are the matrices of the partial derivatives of the mode shapes at the regular S-H sensor points. These matrices are constructed by interpolation from the FE mode shapes.

The matrix

(10) $$J=\left\{\begin{array}{c} \Phi_{x}\\ \Phi_{y} \end{array}\right\}=\left[\begin{array}{cccc} \phi_{1,x} & \phi_{2,x} & \ldots & \phi_{n,x}\\ \phi_{1,y} & \phi_{2,y} & \ldots & \phi_{n,y} \end{array}\right]$$

is the Jacobian of the mode shapes. If the sensor measurements are $s={\left(s_{x}^{T},s_{y}^{T}\right)}^{T}$, the modal amplitudes are solutions of the equation

(11) s=Ja

If the number of active lenslets is $n_{s}$ and the number of modes is *n*, the size of the Jacobian is $\left(2n_{s}\times n\right)$. Assuming that $2n_{s}>n$, the solution is given by the Moore–Penrose pseudo-inverse

(12) $$a=J^{+}\;s$$

which can be readily obtained by singular value decomposition (SVD) [25]. If J is ill-conditioned, the situation may be resolved by one of the following ways: (i) truncating the singular values lower to a threshold in the pseudo-inverse expansion; (ii) using the damped least squares (DLS) method [26].

**Figure 11:**
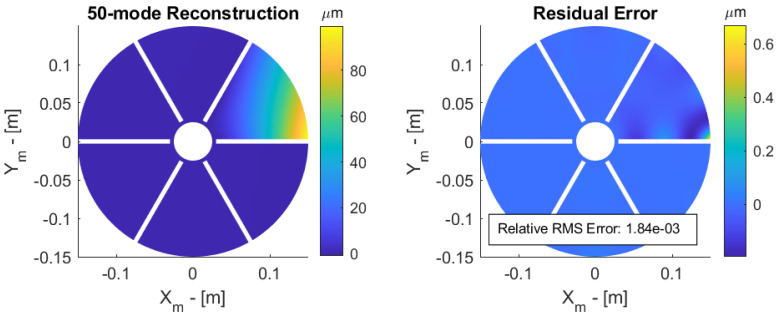
Reconstructed surface (**left**) and residual error (**right**) of a 100 micron tip displacement.

To illustrate the reconstruction method, consider a sensor array of 30 × 30 lenslets; the method is used to reconstruct the tip displacement aberration considered earlier. The reconstructed surface based on slope measurements and the residual error are shown in Figure 11. The RMS residual error is compared with that based on the orthogonality condition in Table 2 for $n_{s}=604$ active lenslets and n=50,100 modes.

### 3.3. Central Obstruction

**Figure 12:**
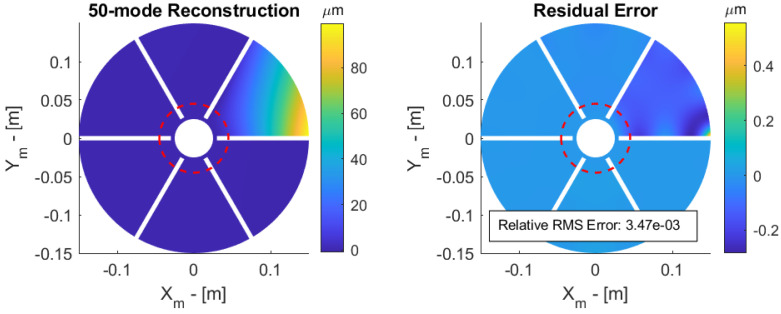
Petal reflector with 30% obstruction. Reconstructed surface (**left**) and residual error (**right**) of a 100 micron tip displacement.

A central obscuration from the secondary mirror is now considered (Figure 8); the central obscuration is defined by the ratio $D_{S}/D_{P}$ between the diameter of the secondary mirror $D_{S}$ and that of the primary mirror $D_{P}$. The petals appear as disconnected when $D_{S}/D_{P}>20\%$; an obstruction of 30% is assumed, so that the petals appear as disconnected. The reduced Jacobian is easily constructed from the full one by considering only the sensor points outside the obstructed area where the measurement vector s is available. Figure 12 shows the reconstructed surface and the residual error outside the obstructed area. The RMS residual error is that shown in the last column of Table 2.

Once the modal expansion of the aberration has been estimated, the *z* displacement can be reconstructed anywhere on the reflector. In particular, reconstructing the displacements on adjacent sides of the petals can provide virtual edge sensors that can be combined with the slope measurements in the shape control of the reflector [1,2,3]. Further studies will be conducted to evaluate the benefit of the procedure for various shapes of the aberration. In particular, the reflector is sensitive to the clamping at the boundary condition, and imperfect clamping leading to sharp transitions may be difficult to recover by the proposed method.

## 4. Conclusions

Two aspects of the metrology of spherical petal reflectors have been addressed:

- The first aspect is that of measuring the surface figure error of a spherical reflector alone, during the development and manufacturing phase. The requested accuracy is modest, because the reflector is intended to be actively controlled once in operation. The SCOTS approach has been found to be fast and satisfactory. Experimental results have been presented and their consistency with a ray-tracing virtual experiment has been assessed.
- The second part of this paper is concerned with the surface figure error reconstruction from slope measurements of a petal reflector when a central part of the mirror is obscured by the secondary mirror of the telescope, making the petals appear as completely disconnected (like segments). Using the fact that all petals have the same mechanical boundary conditions, the deformed shape is expanded in a set of orthogonal modes having the same boundary conditions (the vibration modes). The modal amplitudes are reconstructed from slope data (Shack–Hartmann) and an approximation of the surface figure error is obtained.

## Figures and Tables

**Table 1:** Error between the nominal aberration and the measured aberration including geometric tolerances.

Geometric Change (Camera)	RMS Error
Nominal	0.15 µm
2mm X	0.18 µm
2mm Y	0.17 µm
2mm Z	0.21 µm
2° X	0.65 µm
2° Y	1.18 µm

**Table 2:** Relative RMS error after modal reconstruction based on orthogonality condition (8) and slopes measurements, for 0% and 30% central obstruction with 50 and 100 modes in the reconstruction.

Modes	Equation (8) 0%	Slopes 0%	Slopes 30%
50	1.52 × $10^{-3}$	1.84 × $10^{-3}$	3.47 × $10^{-3}$
100	6.47 × $10^{-4}$	6.47 × $10^{-4}$	6.48 × $10^{-4}$

## Data Availability

Generated data are available upon request. The SCOTS implementation is available in open source [12].

## Abbreviations

The following abbreviations are used in this manuscript:

ESA	European Space Agency
FEA	Finite Element Analysis
FEM	Finite Element Modelling
PV	Peak-to-Valley
PVDF-TrFE	Poly(vinylidene fluoride-co-trifluoroethylene)
RMS	Root Mean Square
SCOTS	Software Configurable Optical Testing System
S-H	Shack–Hartmann
SVD	Singular Value Decomposition

## Appendix A. SCOTS Calculation

The procedure to recover the nine geometric parameters required for the SCOTS calculation [8] of slopes will be explained. It is assumed that the camera aperture location is measured directly, along with an origin point of the screen and mirror, e.g., $x_{c,0}$ for the x-location of the origin of the camera. The location of any point on the mirror is easily recovered under the assumption that the camera image is flat and ignoring the curvature of the mirror:

(A1) $$\begin{array}{cc} x_{m}\left(i,j\right) & =x_{m,0}+s_{camera}\left(j-O_{m,x}\right) \end{array}$$

(A2) $$\begin{array}{cc} y_{m}\left(i,j\right) & =y_{m,0}+s_{camera}\left(i-O_{m,y}\right) \end{array}$$

(A3) $$\begin{array}{cc} z_{m}\left(i,j\right) & =z_{m,0} \end{array}$$

where i,j are the row and column numbers of the pixels in the image and $O_{m,x},O_{m,y}$ are the pixel locations of the center of the mirror in the image. $s_{camera}$ is the camera pixel scale found from the ratio of the diameter of the mirror in pixels, $D_{px}$, and units of length, $D_{mm}$, such that $s_{camera}=\frac{D_{mm}}{D_{px}}$. For simplicity, the operations are carried out over the entire image.

The retrieval of the screen location corresponding to the Mirror Pixel is more involved. First, a series of fringe maps with sinusoidal intensity I are displayed on the screen according to

(A4) $$I_{x}\left(i,j\right)=\frac{A}{2}\sin\left(2\pi \frac{j-O_{s,x}}{P}+\theta \right)+\frac{A}{2}$$

where A is the intensity amplitude, $\left(j-O_{s,x}\right)$ is the pixel distance from the origin, *P* is the amount of pixels per fringe, and θ is a vector of 16 equispaced phase offsets from 0 to 2π. The phase value to be recovered later is $\Phi =2\pi \frac{j-O_{s,x}}{P}$, i.e., θ=0. Several images are taken with different phase offsets and recovered by fitting a sine function to the intensity values of the recovered images in each individual pixel within a pixel-mask of the mirror:

(A5) $${\hat{\Phi }}_{x}\left(i,j\right)={\mathrm{argmin}}_{\Phi }{\left|{\hat{I}}_{x}\left(i,j\right)-\sin\left(\theta +\Phi \right)\right|}^{2}.$$

where ${\hat{I}}_{x}$ is the vector of measured and scaled intensity values with intensity in [−1,1], and θ is the vector of corresponding phase offsets. Note that, fitting in an individual pixel, no global information is present. Therefore, ${\hat{\Phi }}_{x}\left(i,j\right)$ is the wrapped phase, ${\hat{\Phi }}_{x}\left(i,j\right)\in \left[-\pi ,\pi \right]$. The next step is the unwrapping and alignment of the phase. The mirror zero-phase location is found by determining the centroid of an initial image in which only the single pixel corresponding to the screen zero-phase location is lit. The unwrapped phase may then be found by the methods mentioned previously [21,22] (capable of handling the discontinuities).

(A6) $$\Phi_{x}\left(i,j\right)=\mathrm{unwrap}\left({\hat{\Phi }}_{x}\left(i,j\right)\right)+\Phi_{0,x}$$

where $\Phi_{0,x}$ is the measured zero-phase offset. The process to recover the y-phase is equivalent. With this, each Mirror Pixel has corresponding phase values, meaning the screen coordinate can be found:

(A7) $$\begin{array}{cc} x_{s}\left(i,j\right) & =x_{0}+2\pi \Phi_{x}\left(i,j\right)P\;s_{screen} \end{array}$$

(A8) $$\begin{array}{cc} y_{s}\left(i,j\right) & =y_{0}+2\pi \Phi_{y}\left(i,j\right)P\;s_{screen} \end{array}$$

(A9) $$\begin{array}{cc} z_{s}\left(i,j\right) & =z_{s,0} \end{array}$$

where $s_{screen}$ is the screen pixel scale. Now, the geometric vectors can be expressed as follows:

(A10) $$\vec{m2c}=\left(\begin{array}{c} x_{c}-x_{m}\\ y_{c}-x_{m}\\ z_{c}-x_{m} \end{array}\right)\;\vec{m2s}=\left(\begin{array}{c} x_{s}-x_{m}\\ y_{s}-x_{m}\\ y_{s}-x_{m} \end{array}\right)$$

Finally, the slopes can be found according to Equations (1)–(4).
